# Proposing standardised geographical indicators of physical access to emergency obstetric and newborn care in low-income and middle-income countries

**DOI:** 10.1136/bmjgh-2018-000778

**Published:** 2019-07-01

**Authors:** Steeve Ebener, Karin Stenberg, Michel Brun, Jean-Pierre Monet, Nicolas Ray, Howard Lawrence Sobel, Nathalie Roos, Patrick Gault, Claudia Morrissey Conlon, Patsy Bailey, Allisyn C Moran, Leopold Ouedraogo, Jacqueline F Kitong, Eunyoung Ko, Djenaba Sanon, Farouk M Jega, Olajumoke Azogu, Boureima Ouedraogo, Chidude Osakwe, Harriet Chimwemwe Chanza, Mona Steffen, Imed Ben Hamadi, Hayat Tib, Ahmed Haj Asaad, Tessa Tan Torres

**Affiliations:** 1 Health GeoLab Collaborative, Manila, Philippines; 2 WHO Headquarters, Department of Health Systems Governance and Financing (HGF), Geneva, Switzerland; 3 UNFPA, Technical Division, New York City, New York, USA; 4 University of Geneva, Institute of Global Health& Institute for Environmental Sciences, GeoHealth group, Geneva, Switzerland; 5 WHO Regional Office for Western Pacific Region, Manila, Philippines; 6 WHO Headquarters, Department of Maternal, Newborn, Child and Adolescent Health (MCA), Geneva, Switzerland; 7 Freedom Consulting Group, Columbia, Maryland, USA; 8 USAID, Washington, District of Columbia, USA; 9 FHI 360, Durham, North Carolina, USA; 10 WHO Regional Office for Africa, Brazzaville, Congo; 11 WHO Country Office, Manila, Philippines; 12 WHO Country Office, Vientiane, Lao PDR; 13 Ministry of Health, Ouagadougou, Burkina Faso; 14 Pathfinder International, Abuja, Nigeria; 15 Direction générale des études et des statistiques sectorielles, Ouagadougou, Burkina Faso; 16 Pathfinder International, Watertown, New York, USA; 17 WHO Country Office, Lilongwe, Malawi; 18 Geo Expertise, Geneva, Switzerland

**Keywords:** emergency obstetric and newborn care, physical accessibility, universal health coverage, sustainable development goals

## Abstract

Emergency obstetric and newborn care (EmONC) can be life-saving in managing well-known complications during childbirth. However, suboptimal availability, accessibility, quality and utilisation of EmONC services hampered meeting Millennium Development Goal target 5A. Evaluation and modelling tools of health system performance and future potential can help countries to optimise their strategies towards reaching Sustainable Development Goal (SDG) 3: ensure healthy lives and promote well-being for all at all ages. The standard set of indicators for monitoring EmONC has been found useful for assessing quality and utilisation but does not account for travel time required to physically access health services. The increased use of geographical information systems, availability of free geographical modelling tools such as AccessMod and the quality of geographical data provide opportunities to complement the existing EmONC indicators by adding geographically explicit measurements. This paper proposes three additional EmONC indicators to the standard set for monitoring EmONC; two consider physical accessibility and a third addresses referral time from basic to comprehensive EmONC services. We provide examples to illustrate how the AccessMod tool can be used to measure these indicators, analyse service utilisation and propose options for the scaling-up of EmONC services. The additional indicators and analysis methods can supplement traditional EmONC assessments by informing approaches to improve timely access to achieve Universal Health Coverage and reach SDG 3.

**Summary box**

- While it is well known that physical access is a barrier to care-seeking, existing recommended indicators for monitoring the implementation of emergency obstetric and newborn care (EmONC) do not consider travel time required to access health services from a population perspective.

- This paper proposes three geographically explicit indicators to complement the standard set promoted by the international health community for monitoring timely physical access to EmONC: two that measure travel time to EmONC and comprehensive EmONC services, respectively, and a third that focuses on referral time from basic to comprehensive EmONC facilities.

- Adopting the proposed geographically explicit measurements would allow policy makers to monitor timely physical access to essential health care, highlight inequities among the population in terms of travel time and address system barriers on the road to reaching Universal Health Coverage and the Sustainable Development Goals.

## Introduction

Emergency obstetric and newborn care (EmONC) can be life-saving in managing well-known complications during childbirth, but needs to be available, accessible, of quality and used.^1^ Having not achieved these contributed to falling short of Millennium Development Goal (MDG) target 5A^i^. Thus, countries need further efforts to reach Sustainable Development Goal (SDG) target 3.1: reduce the global maternal mortality ratio to less than 70 per 100 000 live births by 2030^ii^.

EmONC services are delivered at two levels of the health system^2^:

1. Basic EmONC (BEmONC) facilities provide seven signal functions or life-saving obstetric services, including administration of (1) parenteral antibiotics, (2) uterotonic drugs and (3) parenteral anticonvulsants for pre-eclampsia and eclampsia; (4) manual removal of the placenta; (5) removal of retained products; (6) assisted vaginal delivery; and (7) neonatal bag and mask resuscitation.
2. Comprehensive EmONC (CEmONC) includes signal functions 1–7 plus (8) caesarean section and (9) blood transfusion.

In low-income and middle-income countries (LMICs), EmONC services are often not universally accessible.^3^ As countries move towards Universal Health Coverage (UHC), timely access to EmONC is critical; thus, assessing timeliness of physical access to EmONC services could help inform national policies.

The global standard is a minimum of five EmONC facilities (including at least one CEmONC facility) per 500 000 population at national and subnational levels. The latter aims to address equity.^2^ However, this indicator does not account for travel time, and thus fails to measure timely access.^4^ It also overlooks that catchment areas of facilities^iii^ are not necessarily confined to administrative boundaries.^5^ Using actual journey time is needed in lieu of straight distance, because the latter fails to reflect real-life travel experience. For example, while 7 of 10 regions of Ghana and 16 of 24 districts in Bangladesh exceeded the recommended target of one CEmONC facility per 500 000 population, most women were unable to access these facilities within safe journey times.^4 6^

Geographical information system (GIS)-based methods applicable to public health^7 8^ and maternal and newborn health,^9^ as well as geospatial data (online supplementary table 1), continue to grow, creating opportunities to use geographically explicit indicators for improved planning and decision making.^4^ This paper presents a set of geographically explicit indicators to estimate physical accessibility to EmONC using case studies from Burkina Faso, Lao People’s Democratic Republic, Malawi and Nigeria. These indicators aim to complement the current standard set of indicators on the availability of EmONC^2^ and address the ‘delay in reaching an adequate health care facility’ from the three delays that affect the interval between the onset of obstetric complication and its outcome.^10^

10.1136/bmjgh-2018-000778.supp1 Supplementary data

## Three indicators to improve timely physical access to emergency obstetric and newborn care

The three indicators described in table 1 are proposed to address two specific questions:

**Table 1:** Proposed indicators for measuring timely physical access to EmONC services

Indicator	Numerator and denominator
ACC1: proportion of pregnant women able to access any EmONC health facility (BEmONC and CEmONC) within a given travel time (unit: %).	Numerator: number of pregnant women residing in a specific geographical area (eg, a country or a district) who are able to access an EmONC health facility (BEmONC or CEmONC) within a given travel time. Denominator: total number of pregnant women residing in the same geographical area.
ACC2: proportion of pregnant women able to access CEmONC health facilities within a given travel time (unit: %).	Numerator: number of pregnant women residing in a specific geographical area who are able to access a CEmONC health facility within a given travel time. Denominator: total number of pregnant women residing in the same geographical area.
REF: proportion of referral linkages between considered BEmONC facilities and their closest CEmONC facility for which the travel time is below a set threshold (unit: %)	Numerator: number of BEmONC health facilities in a specific geographical area within a given travel time of the closest CEmONC facility. Denominator: total number of BEmONC facilities in the same geographical area.

BEmONC, basic EmONC; CEmONC, comprehensive EmONC; EmONC, emergency obstetric and newborn care.

1. Physical accessibility (ACC1 and ACC2): How physically accessible are EmONC services to pregnant women?
2. Referral (REF): How timely is access to EmONC referral facilities?

These indicators have been empirically defined by experts in public health and maternal and newborn health to address gaps they have observed using the current EmONC indicators for real-life situations.

Measurement of these indicators can focus on facilities already providing the signal functions (EmONC facilities) or a ‘designated EmONC network’, which includes EmONC and birthing facilities with potential to provide EmONC once upgraded. The former allows analysing the current effective network, while the latter estimates the potential coverage once the designated EmONC facility network is fully functional. Importantly, the ACC indicators are *population-based*, whereas the REF indicator is *facility-based*.

The input data required to measure the three proposed indicators include (1) the geographical coordinates (latitude, longitude) of health facilities, (2) spatial distribution of pregnant women (target population) in raster format, (3) road and hydrographic networks, (4) digital elevation model, (5) land cover, (6) administrative boundaries, and (7) estimated travel speed on and off the road network (travel scenarios)^iv^. AccessMod V.5.0^v^ and Microsoft Excel are used to determine these geographically explicit indicators. AccessMod, developed and maintained by the WHO since 2005,^11 12^ includes analytical tools to examine physical accessibility to healthcare. It is a free, stand-alone, open source application that can run on Windows, Linux and Mac and process geographical data created by various GIS software (eg, ArcGIS, QGIS and GRASS).

AccessMod has been used to examine physical accessibility and geographical coverage taking into account the spatial distribution of the population and the health system capacity to serve this population.^13–19^ Recent applications^20–27^ have assessed physical accessibility to EmONC services or tested different scenarios to optimise the EmONC facility network (eg, establishing new facilities or maternity waiting homes,^28^ selecting facilities for upgrade, and improving motorised vehicles access after constructing, repairing, or enhancing roads and bridges).

Accessibility modelling in AccessMod is based on a least-cost path algorithm that minimises travel time between any two locations and allows assessing physical access across administrative borders. The proposed accessibility-based indicators are standardised by using similar algorithms (described in detail in ref 12), but the number, quality and sources of the input data sets can vary between countries owing to data availability.

Building accessibility models that factor in various modes of transport requires assuming (1) the combination of transport modes (eg, walking to the nearest road and then using a motorised vehicle), (2) the average speed of travel on different types of road or land cover, and (3) the appropriate maximum travelling time. These assumptions and associated uncertainties can vary between countries and target populations. Testing alternative models can clarify the impact of these uncertainties and trigger further investigations to improve the models.

Finally, while travel time fluctuates with time of day and seasonality, only weather conditions that can be captured geographically (ie, flooding) are considered here.

### Physical accessibility coverage indicators

The first two proposed indicators, which assess physical accessibility of pregnant women to health facilities (EmONC facilities or the designated EmONC network), are defined (table 1):

- ACC1: proportion of pregnant women able to access any EmONC health facility (BEmONC or CEmONC) within a given travel time.
- ACC2: proportion of pregnant women able to access CEmONC health facilities within a given travel time.

Both indicators are measured by dividing the number of pregnant women residing in a specific geographical area that can access the considered type of facility within a given travel time, by the total number of pregnant women in that geographical area.

These indicators should be measured at the subnational level to identify inequities that can be masked using national figures. Comparing results with specific national or international UHC targets can help in decision making (eg, at least 90% of all births attended by skilled birth attendants as agreed in 2015 in the International Conference on Population and Development^29^).

The value for these indicators can be obtained in AccessMod. First, the geospatial data defining the environment being travelled (eg, road network, land cover, hydrography), the health facility locations and the travel scenario are used in accessibility analysis modules to obtain the spatial distribution of the travel time to the closest EmONC facility. The resulting travel time distribution, the geographical distribution of the population and national administrative boundaries are entered into the zonal statistics module to generate the ACC indicator values for a specific travel time.

The maximum suggested travel time is 2 hours based on clinical studies that identified this as being the average time between onset of untreated severe postpartum haemorrhage (a leading cause of maternal death) and death.^30^ Furthermore, empirical studies show successively increased obstetric case-fatality rates with journey times greater than 2 hours.^31^

The detailed data and assumptions used and the process followed by AccessMod can be found in the user manual and reports.^20–22^

### Referral indicator

The third proposed indicator reflects the referral network capacity to provide effective transfer between the considered BEmONC and CEmONC facilities (table 1):

- REF: proportion of referral linkages between considered BEmONC facilities and their closest CEmONC facility for which the travel time is below a set threshold.

This indicator is measured by dividing the number of BEmONC facilities within a set travel time to their nearest CEmONC facility, by the number of BEmONC facilities in the covered geographical area.

All considered EmONC facilities in the study area are taken into account when measuring this indicator; thus, the nearest CEmONC might be located in a neighbouring subnational division.

The maximum referral travel time is recommended to be 2 hours given that most postpartum haemorrhage deaths occur within 2 hours of onset.^32^

Referral between a BEmONC and a CEmONC facility is dependent on functional and available communication and transportation. Without ready transportation, delays occur in hiring a private car or contacting other facilities to send a vehicle. Unavailability of functioning communication between health facilities (eg, phone, radio) also prolongs referral times. These constraints should be considered when measuring this indicator.

Calculating the referral indicator requires obtaining the travel time between each BEmONC and its nearest CEmONC. AccessMod has a referral module to facilitate this analysis.^21 22^ These travel time values are then adjusted to account for available transportation and communication.

## Supplementary analyses to inform emergency obstetric and newborn care policies

We provide here examples applying the proposed indicators to inform EmONC policies.

### Service utilisation analysis

Physical accessibility alone does not imply that services are sufficient to cover the demand or that services are used. This requires supplementary analyses.

Service utilisation can be assessed overall at the national level, or by measuring the actual coverage of each EmONC facility’s catchment area. A facility-specific measure of actual coverage may be obtained for an EmONC facility by dividing the total births (live births and stillbirths) that occur at each facility among women living in the catchment area by the total number of expected births (pregnant women) for that catchment area. This complementary measure is different from the ‘Met need for emergency obstetric care’ indicator,^2^ which considers only women with major direct obstetric complications who sought facility-based care and women expected to have those complications.

Ideally, while avoiding ethical breaches, the geographical location of the lowest administrative unit of residence (eg, village) of each pregnant woman delivering in an EmONC facility is collected. This allows identification of women travelling from another catchment area. Unfortunately, these data are not usually available in LMICs. Alternatively, proxy measures for this actual coverage can be obtained from surveys with household-level data (eg, Demographic and Health Surveys (DHS)). However, these surveys neither allow differentiation between births that take place in an EmONC facility versus other facilities nor are they representative at the facility catchment area level.

Actual EmONC coverage is also influenced by the capacity of the EmONC network to cover the demand. Measures of capacity may consider the estimated maximum coverage based on the available skilled birth attendants and other availability or quality of care-related factors. Incorporating such measures into the physical accessibility analysis allows measuring geographical coverage.

The AccessMod geographical coverage module can be used to measure this concept and therefore identify areas where augmenting service capacity would enable the expansion of EmONC coverage. However, this indicator is not promoted for inclusion into the current standard indicators due to difficulties in obtaining data needed to estimate the maximum coverage capacity of each considered health facility.

Service utilisation analysis is conducted by graphically comparing subnational values for the ACC indicators with the corresponding geographical coverage measures and actual coverage. Such analysis accounts for the association of time travelled and utilisation, and helps determine whether availability or physical accessibility is the greater barrier to EmONC service utilisation, while considering that women living close by are more likely to use them.

Additional data needed to conduct the service utilisation analyses include subnational service utilisation, health facility births or the national standard workload per skilled birth attendant. A description on how to conduct such analysis in AccessMod is available in several reports.^23–26^

### Scaling-up analysis

To test different scenarios designed to increase access to care for the population within a given travel time, we use the ACC and REF indicators in a scaling-up analysis. This analysis can also be conducted to model improved performance for the service utilisation analysis.

AccessMod modules can be used to test scenarios for investments to expand access to the healthcare facility network, by:

1. Upgrading birthing facilities to become EmONC facilities.
2. Building new roads or bridges to increase transportation efficiency.
3. Improving the availability of transportation and communication technology at facilities’ disposal to reduce referral travel time.
4. Identifying the most suitable locations for additional EmONC facilities.
5. Expanding the capacity of existing facilities and/or establishing maternal waiting homes (MWHs).

Data needed to apply these scenarios include the geographical location of the considered infrastructure (eg, health facilities, roads, bridges) and the capacity of these same health facilities.

## Empirical applications of the indicators

We present examples from Burkina Faso, Lao People’s Democratic Republic, Malawi and Nigeria, where the proposed indicators have been measured and complementary analyses carried out using AccessMod.

Online supplementary table 1 provides the format, source and common limitations when looking for available quality data across the empirical studies covered here.^8 9^ The difference in sources observed between countries is linked to the limitations mentioned above, with priority given to available national data over regional or global data sets.

All country results presented here consider the standard definitions for BEmONC and CEmONC during the dry season, and assume that women would seek care during early labour by walking or being carried to the nearest road where a motorised vehicle would take her to a facility.

However, variation exists between countries regarding the health facilities in the analysis (actual or designated EmONC facilities), the travel speed used per mode of transportation and land cover/road type. The country-specific studies provide greater detail on these assumptions.^20–26^

### Physical accessibility coverage—Malawi

The physical accessibility analysis in Malawi was based on health facility data collected during the 2010 EmONC needs assessment.^33^ It allows categorisation of the ACC1 indicator (EmONC facilities) to the district level for 1, 2, 3 and 4 hours of travel time. It did not consider additional birthing facilities with EmONC potential.^20^ In 2010, the density of EmONC facilities per 500 000 was 1.69 out of 5 at the national level, which corresponds to 33.8% of the recommended minimum number of EmONC facilities.

Table 2 presents the results obtained for this indicator at the national, regional and district levels. The results are representative of the situation observed in 2010 and point to a 90% EmONC physical accessibility coverage for pregnant women living between 2 and 3 hours of travel time from an EmONC facility. This was true at the national level and for both Central and Southern Regions; however, 90% EmONC physical accessibility coverage is obtained only between 3 and 4 hours of travel time for the Northern Region. Similar variability is seen with districts within regions, illustrating how aggregated figures mask pockets of heterogeneity in healthcare access at lower levels. Therefore, to ensure equity in the advancement towards UHC, physical access needs to be addressed at the lowest possible level of geographical disaggregation.

**Table 2:** National-level, regional-level and district-level percentages of pregnant women within a given travel time to the nearest emergency obstetric and newborn care facility—Malawi (extracted from ref 20)*

Region name	District name	ACC1 (1 hour)		ACC1 (2 hours)		ACC1 (3 hours)		ACC1 (4 hours)	
		Region (%)	District (%)	Region (%)	District (%)	Region (%)	District (%)	Region (%)	District (%)
Central Region	Dedza	69.5	64.9	89.7	90.3	95.4	95.8	97.7	98.3
	Dowa		66.1		90.5		96.7		99.0
	Kasungu		48.3		74.1		85.4		92.2
	Lilongwe		86.4		96.4		98.7		99.4
	Mchinji		65.1		88.7		95.4		97.9
	Nkhotakota		41.8		82.6		93.3		96.5
	Ntcheu		61.6		87.0		94.5		97.0
	Ntchisi		65.9		89.7		95.7		97.7
	Salima		73.4		91.6		96.0		97.1
Northern Region	Chitipa	53.1	48.5	79.6	76.2	89.2	87.0	94.2	91.3
	Karonga		70.2		87.3		91.8		94.8
	Likoma		0.0		0.0		9.1		99.7
	Mzimba		56.3		81.7		91.5		96.0
	Nkhata Bay		47.0		73.7		83.3		88.1
	Rumphi		27.7		73.7		88.4		94.8
Southern Region	Balaka	64.7	66.5	88.9	92.1	95.7	97.6	97.9	99.0
	Blantyre		90.6		97.9		99.6		99.9
	Chikwawa		53.6		80.3		91.2		94.5
	Chiradzulu		87.5		99.5		100.0		100.0
	Machinga		45.5		85.0		96.5		99.1
	Mangochi		52.5		81.0		90.9		95.3
	Mulanje		73.6		91.7		95.7		96.8
	Mwanza		64.5		88.2		94.8		98.0
	Neno		57.0		85.6		94.5		98.0
	Nsanje		15.3		76.3		91.4		96.8
	Phalombe		67.5		94.1		98.4		99.5
	Thyolo		54.4		80.3		91.7		96.6
	Zomba		73.0		96.1		99.4		99.9
	Nationwide	65.2		88.0		94.7		97.3	

*Values above 90% are highlighted in green.

ACC, accessibility coverage; REF, referral.

### Referral—Nigeria

The Saving Mothers, Giving Life (SMGL) initiative supported a study aimed to determine the accessibility to EmONC facilities among local government areas in Cross River State for 2015.^21^ The study included the analysis of referral time between each of the 116 potential BEmONC and 19 CEmONC facilities selected to become functional through SMGL’s interventions among existing public and private facilities. Of note, the densities of selected EmONC and CEmONC facilities per 500 000 populations were 18.5 and 2.6, respectively, both above the international benchmark.^2^

**Figure 1:**
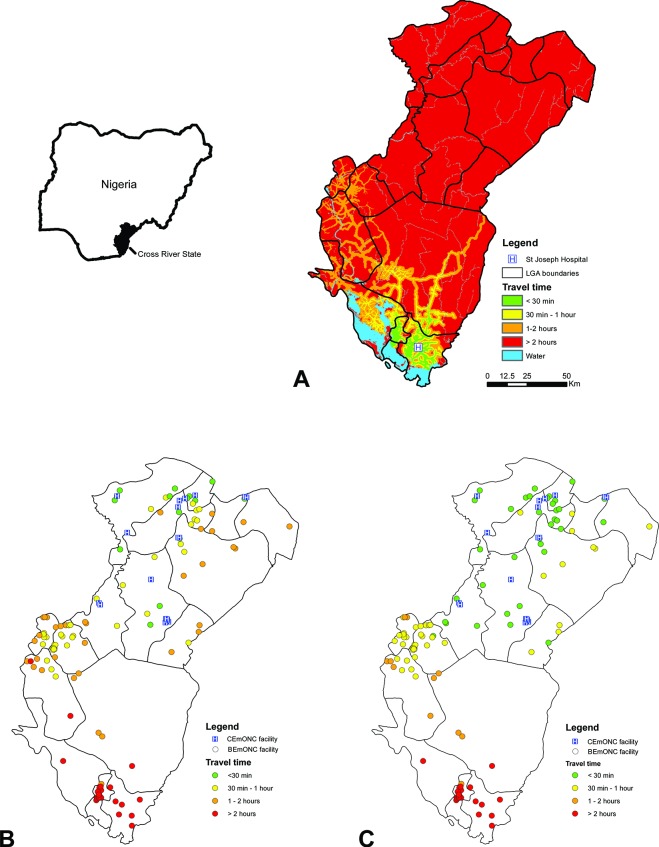
(A) Example of a travel time distribution grid from one BEmONC facility (St Joseph Hospital): travel time between each BEmONC facility to the nearest CEmONC facility when considering (B) the current situation in terms of availability of a functioning motor vehicle and mode of communication, and (C) the hypothetical situation where all BEmONC facilities have a functioning motor vehicle on site, Cross River State, Nigeria.^21^ BEmONC, basic EmONC; CEmONC, comprehensive EmONC; EmONC, emergency obstetric and newborn care; LGA, local government area.

The referral analysis module of AccessMod was used to measure the travel time (in minutes) between BEmONCs and the nearest CEmONC facility. Figure 1A illustrates the travel time distribution for the BEmONC St Joseph Hospital and the 19 nearest CEmONC facilities.

The availability of a functioning motor vehicle and communication mode at the BEmONC site was taken into account to adjust the original travel time using the following assumptions (modified from a similar exercise^34^):

1. If the BEmONC facility has a motorised vehicle on site, the transfer time is equal to the direct travel time to the nearest CEmONC facility.
2. If the BEmONC facility does not have a motorised vehicle, but has access to communication such as a phone:
  - The BEmONC facility contacts the closest facility having a motor vehicle and a phone.
  - The vehicle comes from that facility and transports the patient to the closest CEmONC facility.
  - The total referral time equals the travel time between the facility contacted and the BEmONC facility, and the travel time between the BEmONC facility and the nearest CEmONC facility.
3. If the BEmONC facility has neither a motorised vehicle nor a means of communication on site, the same approach as in assumption 2 is considered but an additional 30 min is added to the calculation to account for the notification and response time.

Figure 1B shows 88 of the 108 birthing facilities with EmONC potential selected in the northern part of Cross River State are located within less than 2 hours of travel time of a CEmONC facility. This corresponds to an REF indicator value of 81.5%. This contrasts with the southern part of the State, where 25 of the 27 birthing facilities are located more than 2 hours away (REF of 7.4%). Ensuring a motorised vehicle is available would solve the issue for only 2 of these 25 BEmONC facilities (figure 1C).

These results led SMGL to select additional CEmONC facilities in the southern part of the State, an adjustment that would not have occurred had they looked only at the density of EmONC facilities available.

### Service utilisation analysis—Burkina Faso

**Figure 2:**
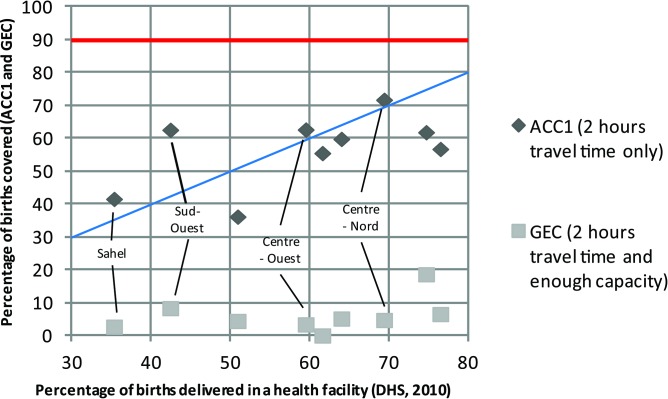
Region-level percentage of births covered by emergency obstetric and newborn care facilities as determined by the accessibility (ACC1) and the geographical coverage analysis (GEC) plotted against the percentage of births delivered in a public or private health facility in the preceding 5 years, Burkina Faso (modified from ref 23). DHS, Demographic and Health Survey.

Accessibility to emergency obstetric care and geographical coverage was examined in Burkina Faso.^23^ Service utilisation was analysed in figure 2 by comparing subnational figures for the ACC1 indicator and the geographical coverage measure for 2 hours of travel time plotted against the percentage of health facility live births in the 5 years preceding the 2010 DHS.^35^

All regions have ACC1 indicators and the geographical coverage values below a 90% benchmark (red line in figure 2). This indicates that both availability of services and physical accessibility are barriers to EmONC utilisation in Burkina Faso. Furthermore, Sahel, Sud-Ouest, Centre-Ouest and Centre-Nord Regions present an ACC1 indicator value higher than the percentage of births delivered in a health facility (above the blue line in figure 2). This may indicate that the number of BEmONC facility births is higher in those regions than the other five. Finally, geographical coverage is much lower than births taking place in a facility, which indicates that availability is a more significant barrier than accessibility in regard to BEmONC services.

### Scaling-up analysis—Lao People’s Democratic Republic

Two scaling-up scenarios aimed at increasing geographical coverage to EmONC were tested in the Lao People’s Democratic Republic^26^:

- Scenario 1: expanding the existing network of EmONC facilities (18 facilities) to 83 facilities per the national EmONC improvement plan^36^ and the staffing capacity of these facilities to be able to service all births for households located within 2 hours of travel time.
- Scenario 2: establishing an MWH near each of the existing 18 EmONC facilities and modelling the capacity that would be required to accommodate all women living between 2 and 6 hours of travel time from these facilities to access these homes prior to giving birth.

Geographical coverage before and after applying the two scenarios in AccessMod are presented in table 3. Increasing the number of EmONC facilities as proposed in the improvement plan (scenario 1) would increase national geographical coverage by 66.3%, but inequalities would remain at the subnational level. Building MWHs (scenario 2) would further increase coverage by 3.1% but would impact provinces differently compared with the first scenario; inequities would still remain.

**Table 3:** Province-level geographical coverage before the scaling-up and after applying the two scenarios for basic emergency obstetric and newborn care facilities—Lao People’s Democratic Republic (modified from ref 26)

Province name	Geographical coverage before the scaling-up (%)	Geographical coverage after applying the first scenario (%)	Geographical coverage after applying the second scenario (%)
Attapu	0.0	81.0	87.5
Bokeo	0.0	79.6	56.0
Bolikhamxai	44.5	96.8	98.8
Champasak	22.3	95.6	98.5
Houaphan	9.9	70.5	78.8
Khammouan	22.0	98.1	99.7
Louang-Namtha	0.0	68.8	74.4
Louangphabang	0.3	76.4	83.5
Oudomxai	0.0	58.9	71.9
Phongsali	31.0	76.2	89.5
Salavan	6.9	96.1	99.5
Savannakhet	11.4	97.6	99.6
Xekong	0.0	87.7	94.1
Vientiane	48.2	93.9	98.5
Vientiane Capital	99.2	100.0	100.0
Xaignabouli	0.0	90.6	92.0
Xiangkhouang	31.8	97.1	98.7
National	23.5	89.8	92.9

Planners can use results like these to estimate the investments needed. In the Lao People’s Democratic Republic case, the first scenario would require relocating 3272 skilled birth attendants. The second scenario would require relocating 1271 skilled birth attendants and construction and maintenance of 18 MWHs.

## Addressing limitations of the proposed indicators

Limitations for measuring, using and interpreting the three indicators described here exist. This is especially true in the LMICs most in need of improving timely physical access and addressing inequities.

The first limitation is the availability, quality and accessibility of the required data sets.^9^ While there is growing interest in geospatial technologies (including GIS, remote sensing and global navigation satellite systems) across LMICs, having access to a complete, up-to-date and georeferenced health facility master list remains a challenge. Other environmental data such as land cover can be obtained from free global data sets in case no country-specific data are available (online supplementary table 1); however, these data sets are often outdated, may require substantial cleaning and are usually limited to analysis representative of better conditions observed during the dry season. Performing the same analysis for the wet season would likely show worse conditions. The subnational percentage of health facility live births needed for the service utilisation analysis may be accessible through censuses or household surveys such as the DHS; however, these are often conducted with several years interval in between and may not be available in areas inflicted with war or famine.

The second limitation concerns access to recent and comprehensive EmONC assessments that provide the complete list of considered health facilities (EmONC facilities or designated EmONC network), information about the quality of care and current system capacity. The latter includes availability of health workers, and the number of attended deliveries and caesarean sections. The use of old or partial assessment data may result in an underestimation of the proposed indicators. Also, many country EmONC assessments do not include private sector facilities.

To facilitate the collection of EmONC data, the United Nations Population Fund’s (UNFPA) has developed a lighter EmONC assessment tool^vi^. This has been implemented in Ivory Coast, Niger, Mali, Mauritania and in Eastern Africa. In addition, the UNFPA is supporting Benin, Burundi, Guinea (Conakry), Madagascar, Senegal and Togo to define their national EmONC facility networks. For this work, AccessMod has been used to strengthen network functioning by implementing a process to monitor obstetric activity, staff skills and the quality of network referral links, and to proactively respond to gaps in quality of care.

Even when data are available, temporal discrepancies often exist between data sets. While the timing of survey/census data collection is generally available, this is not always the case with geographical data and it is sometimes difficult to assess these discrepancies. For example, sometimes available data sets are outdated. Another limitation is availability of local technical expertise to use geographical data and related technologies.^9^ Such expertise is required to prepare input data, apply tools such as AccessMod, and critically evaluate and analyse the results generated.

To address the above limitations, international public health and GIS experts are closely collaborating with national programmes to use geographical data and technologies for maternal and newborn health,^9^ as they do with other programmes such as immunisation.^37^ The integration of skills and tools should lead to improved availability, quality, accessibility and utilisation of data.

Another limitation in using the proposed indicators is their lack of clear targets. The REF target should be set to 100% for a 2-hour travel time to avoid the high risk of mortality beyond this period; however, setting a target for the ACC indicators is less straightforward as the scientific evidence is in an early stage of evolution and thus needs further research.

Additionally, these indicators are designed to inform where and how best to deliver care for women with obstetric emergencies. In contexts that depend heavily on MWHs located near functioning EmONC facilities, these indicators may be less informative or misleading as they would not account for the existence of such infrastructures.

Lastly, the proposed indicators focus on physical accessibility and not on the quality of care provided. Quality is captured through other indicators outside the scope of this paper.

## Conclusion

This paper proposes three geographically explicit indicators for widespread adoption: (1) proportion of pregnant women able to access EmONC health facilities (BEmONC or CEmONC) within a given travel time; (2) proportion of pregnant women able to access CEmONC health facilities within a given travel time; and (3) proportion of referral linkages between the considered BEmONC facilities and their nearest CEmONC facility for which the travel time is below a set threshold. Expanding standard monitoring processes for EmONC to include these indicators would strengthen information on supply-side systems capacity and on equity gaps in access to care and could thus lead to improved policy making.

Countries can use these indicators and associated analyses to assess whether EmONC services are physically accessible to pregnant women. This can guide strategy, plan and programme development towards reaching UHC and reducing maternal and newborn mortality by 2030 under the umbrella of the SDGs.

Five specific activities are anticipated as the way forward:

1. Further validate the indicators and recommend potential targets through additional incountry studies.
2. Demonstrate the value added for national planning processes through country case studies.
3. Encourage the addition of these indicators to the current standard set for monitoring EmONC and incorporation of this into updates of the Monitoring Emergency Obstetric Care Handbook.^2^
4. Continue improving data quality and increasing the availability of data that take into account seasonal variation and place of residence.
5. Strengthen national and regional technical capacity to measure, analyse and use the proposed set of geographically explicit EmONC indicators.

Ongoing projects supported by the Asian Development Bank in Myanmar and Cambodia, and by the UNFPA in Benin, Burundi, Guinea (Conakry), Madagascar, Senegal and Togo, will help validate the indicators and potentially recommend targets.

Improvements of data availability, quality and accessibility and incountry technical capacity strengthening are expected to be covered through the establishment of regional hubs. This is already occurring in some regions of the world. For ‘’example, the Health GeoLab Collaborative, formerly AeHIN GIS Lab,^8 38^ has been established to provide more technical support to countries in Asia and the Pacific and could be replicated in other regions.

## Footnotes

The MDG 3 target 5A called for a reduction of at least 75% in the maternal mortality ratio (MMR), but the global reduction in MMR fell by only 44% between 1990 and 2015.^39^

https://sustainabledevelopment.un.org/sdg3.

Geographical area from which a health facility attracts a population that uses its services.

Definitions for the GIS-specific terms used in this paper can be found in the following online dictionary: http://www.wiki.gis.com/wiki/index.php/GIS_Glossary.

https://www.accessmod.org.

https://wcaro.unfpa.org/fr/publications/soins-obst%C3%A9tricaux-et-n%C3%A9onataux-durgence
