# Supplementary material for: Proposing standardised geographical indicators of physical access to emergency obstetric and newborn care in low-income and middle-income countries
Source: BMJ Glob Health. 2019 Jul 1;4(Suppl 5):e000778. doi: 10.1136/bmjgh-2018-000778 (PMC6623986; doi:10.1136/bmjgh-2018-000778)
Supplement: Supplementary data [file bmjgh-2018-000778supp001.pdf]

Table S1 Type, format, source and main limitations encountered for the data used in analysis presented in the paper

Data (type, format)	Burkina Faso	Lao PDR	Malawi	Nigeria (Cross River State)	Main data limitations encountered during the study
Administrative division boundaries (geospatial, vector format)	Geographic Institute of Burkina Faso through the SALB project [1]	Lao DECIDE Info online platform [2]	Department of Survey of Malawi through the SALB project [1]	Office of the Surveyor General of the Federation (OSGOF) through the SALB project [1]	Data quality (timeliness, accuracy)
List and geographic coordinates of the EmONC facilities (geospatial, vector) as well as attribute data (tabular, excel format)	2010 EmONC needs assessment [3]	Combination of different sources of information [4]	2010 EmONC needs assessment [5] and Ministry of Health of Malawi	2012 Rapid Health Facility Assessment [6] and Nigeria MDG Information System (NMIS) [7]	Data availability; Data quality (accuracy, completeness)
Land cover distribution (geospatial, raster format)	Global Mapping project [8] combined with the extent of urban areas from the Global Rural-Urban Mapping Project (GRUMP) [9]			Global Land Cover Dataset (GLobeLand30) [10]	Data handling (reclassification)
Road network (geospatial, vector format)	OpenStreetMap [11]	Lao National Geographic Department (NGD)	Department of Surveys of Malawi and the National Road Authority of Malawi	OpenStreetMap [11]	Data handling (cleaning, reclassification); Data completeness
Hydrographic network (geospatial, vector format)	Global Insight plus [12]	Lao National Geographic Department (NGD)	Department of Surveys of Malawi	OpenStreetMap [11]	Data accessibility (Proprietary); Data handling (cleaning); Data completeness
Digital Elevation Model (DEM) (geospatial, raster format)	1 sqm Shuttle Radar Topography Mission (SRTM) dataset [13]		90m SRTM dataset [13]		None
Target population distribution (Geospatial, raster format)	2008 Landscan database [14] adjusted to subnational number of births for 2010 [15]	2008 Landscan database [14] adjusted to subnational number of births for 2011 [4]	WorldPop [16] adjusted to subnational number of pregnant women for 2010 [17-18]		Data availability (subnational level figures); Data accessibility (proprietary)
Maximum acceptable workload for skilled birth attendant (norm)	WHO [19] adjusted to the local context [20]	2008-2012 Skilled Birth Attendance Development Plan [21]	Not applicable	Not applicable	Data availability and level of desegregation
Service utilisation (tabular, Excel format)	2010 Demographic Health Survey [15]	2011-12 Lao Social Indicator Survey [22]	Not applicable	Not applicable	Data availability and level of desegregation
Travelling scenario (tabular, Excel format)	WHO [23] adjusted with local information [20]	WHO [23] adjusted with local information [4]	WHO [23] adjusted with local information [17]	WHO [25] adjusted with local information [18]	Data availability

Table S1 Type, format, source and main limitations encountered for the data used in analysis presented in the paper

1 unsalb.org [Internet]. Second Administrative Level Boundaries (SALB) dataset web site [cited 2018 Jun 12]. Available from: https://www.unsalb.org/.

2 decide.la [Internet]. Lao DECIDE Info online platform [cited 2018 Jun 12]. Available from: http://www.decide.la.

3 Centre Nationale de la Recherche Scientifique et Technologique. Evaluation des Besoins en Soins Obstétricaux et Néonataux d'Urgence, couplée à la cartographie de l'offre de soins en Santé de la Reproduction au Burkina Faso. Ministère de la Recherche Scientifique et de l'Innovation, 2011.

4 World Health Organization. Investing the Marginal Dollar for Maternal and Newborn Health: Geographic Accessibility Analysis for Emergency Obstetric Care services in Laos. WHO Report 2016 [cited 2018 Jun 12]. Available from: http://apps.who.int/iris/bitstream/10665/250273/1/WHO-HIS-HGF-GIS-2016.4-eng.pdf.

5 Malawi Ministry of Health: Malawi 2010 EmONC Needs Assessment. Final Report, Lilongwe, Feb 2011.

6 Cross River State Ministry of Health and FHI 360. 2013. Cross River State-wide Rapid Health Facility Assessment, Nigeria: Cross River State Ministry of Health and FHI 360. Final report 2013 [cited 2018 Jun 12]. Available from: https://www.fhi360.org/sites/default/files/media/documents/Cross%20River%20RHFA%20report.pdf.

7 Center for Sustainable development, Nigeria MDG Information System: (NMIS) Takes Open Data Further. Earth Institute, Columbia University. 2014 March 10 [cited 2018 Jun 12]. Available from: http://csd.columbia.edu/2014/03/10/the-nigeria-mdg-information-system-nmis-takes-open-data-further/.

8 https://globalmaps.github.io/ [Internet]. Global Map data archives [cited 2018 Jun 12]. Available from: https://globalmaps.github.io/.

9 Center for International Earth Science Information Network (CIESIN)/Columbia University, International Food Policy Research Institute (IFPRI), The World Bank, and Centro Internacional de Agricultura Tropical (CIAT). 2011 [cited 2018 Jun 12]. Global Rural-Urban Mapping Project, Version 1 (GRUMPv1): Urban Extents Grid. Palisades, NY: NASA Socioeconomic Data and Applications Center (SEDAC). Available from: http://sedac.ciesin.columbia.edu/data/set/grump-v1-urban-extents.

10 Global Land Cover Dataset (GLOBELAND30) [Internet]. [cited 2018 Jun 12]. Available from http://www.globeland30.org/GLC30Download/index.aspx .

11 OpenStreetMap [Internet]. [cited 2018 Jun 12]. Available from http://www.openstreetmap.org/.

12 Global insight plus [Internet]. Europa Technologies [cited 2018 Jun 12]. Available from https://www.europa.uk.com/map-data/global-map-data/global-insight-plus/.

13 Shuttle Radar Topography Mission [Internet]. Jet Propulsion Laboratory, California Institute of Technology Data Products: SRTM C-Band Data Products [cited 2018 Jun 12]. Available from: http://www2.jpl.nasa.gov/srtm/cbanddataproducts.html.

14 LandScan: Geographic Information Science and Technology [Internet]. Oak Ridge National Laboratory [cited 2018 Jun 12]. Available from: http://web.ornl.gov/sci/landscan/.

15 Institut National de la Statistique et de la Démographie (INSD) et ICF International. Enquête Démographique et de Santé et à Indicateurs Multiples du Burkina Faso 2010. Calverton, Maryland, USA : INSD et ICF International 2012 [cited 2018 Jun 12]. Available from: https://www.dhsprogram.com/pubs/pdf/FR256/FR256.pdf.

16 WorldPop [Internet]. [cited 2018 Jun 12]. Available from: http://www.worldpop.org.uk/.

17 USAID. Malawi - Accessibility to EmOC facilities. USAID report 2015 [cited 2018 Jun 12]. Available from: http://www.healthgeolab.net/PUB/USAID_MWI_2015.pdf

18 USAID. Nigeria - Accessibility to EmOC facilities in the State of Cross River. USAID report 2016 [cited 2018 Jun 12]. Available from: https://pdf.usaid.gov/pdf_docs/pa00mdwb.pdf .

19 World Health Organization. The World Health Report 2005 - make every mother and child count. Geneva, 2005 [cited 2018 Jun 12]. Available from: http://www.who.int/whr/2005/en/.

20 World Health Organization. Investing the Marginal Dollar for Maternal and Newborn Health: Geographic Accessibility Analysis for Emergency Obstetric Care services in Burkina Faso. WHO Report 2016 [cited 2018 Jun 12]. Available from: http://apps.who.int/iris/bitstream/10665/250269/1/WHO-HIS-HGF-GIS-2016.1-eng.pdf.

21 Lao People's Democratic Republic Ministry of Health (2009): Skilled Birth Attendance Development plan Lao PDR 2008-2012

22 Ministry of Health/Lao PDR and Lao Statistics Bureau. 2012. Lao Social Indicator Survey 2011-12. Multiple Indicator Cluster Survey/Demographic and Health Survey. Vientiane, 2012 Dec [cited 2018 Jun 12]. Available from: https://dhsprogram.com/pubs/pdf/fr268/fr268.pdf.

23 World Health Organization. Global status report on road safety: time for action. Geneva, 2009 [cited 2018 Jun 12]. Available from: http://www.who.int/violence_injury_prevention/road_safety_status/2009/en/.

25 World Health Organization. Global status report on road safety 2013: Supporting a decade of action. Geneva, 2013 [cited 2018 Jun 12]. Available from: http://www.who.int/violence_injury_prevention/road_safety_status/2013/en/.
